# Maturational Hyperpigmentation: Cutaneous Marker of Metabolic Syndrome

**DOI:** 10.5826/dpc.1002a46

**Published:** 2020-04-20

**Authors:** Sidharth Sonthalia, Mahima Agrawal, Poonam Sharma, Amarendra Pandey

**Affiliations:** 1 SKINNOCENCE: The Skin Clinic & Research Center, Gurugram, India; 2 LHMC & Associated Hospitals, New Delhi, India; 3 Skin Institute & School of Dermatology (SISD), New Delhi, India; 4 Cosmasure Skin Hair, Fat Management and Laser Clinic, Jabalpur, India

**Keywords:** maturational hyperpigmentation, facial melanosis, metabolic syndrome, facial acanthosis nigricans, dermoscopy

## Case Presentation

**Figure 1:**
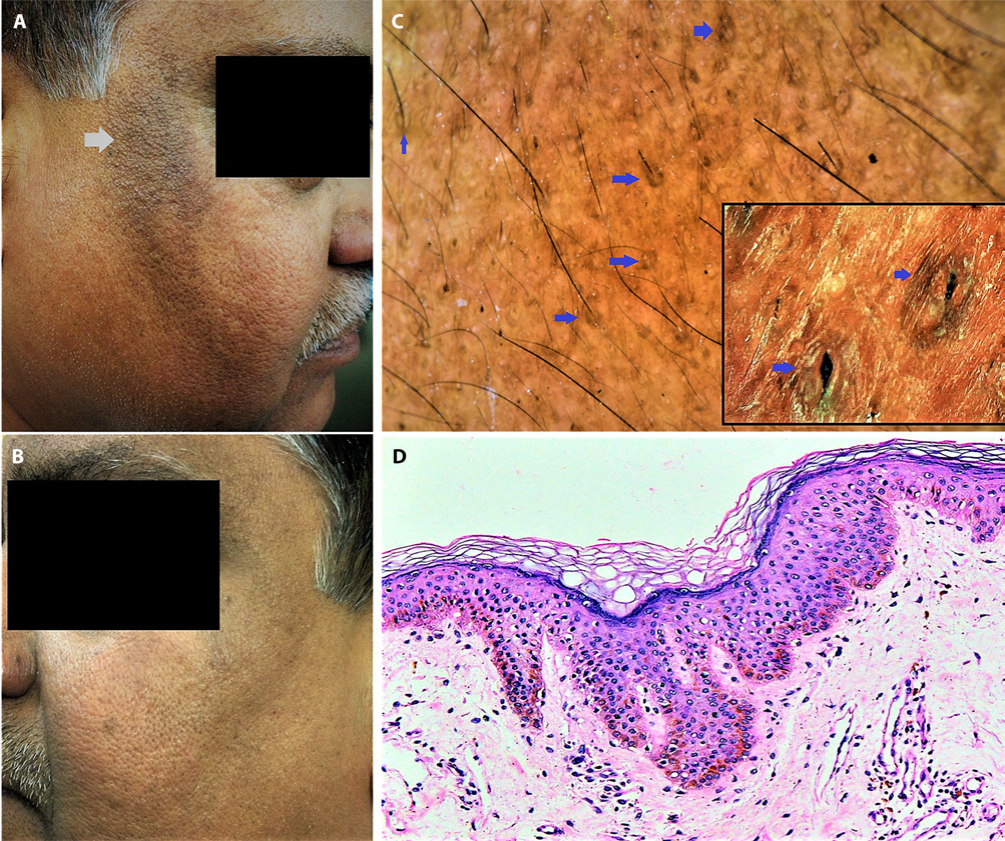
Maturational hyperpigmentation. (A) Patch of dark brown pigmentation with ill-defined margins and conspicuous granular surface over the right cheek (white arrow) extending from the angle of the eye to nearly the angle of the mouth. (B) Similar but much lighter pigmentation over the left cheek suggestive of an evolving lesion. (C) Videodermoscopic image of the hyperpigmentation on the right side revealing exaggerated light brown pseudoreticular network with scattered brown globules and structureless areas, perifollicular oval dark brown “rings” (blue arrows), and absence of sulci and cristae, the dermoscopic features that are pathognomonic of cutaneous acanthosis nigricans. The perifollicular rings can be appreciated better at higher magnification (blue arrows) in the figure inset (polarized, ×35 and ×150; Escope Videodermoscope, Timpac Healthcare Pvt. Ltd., New Delhi, India). (D) Histopathology showing basal cell layer melanocytosis, hypermelanization of basal and squamous layers of epidermis, and the dermis. Hyperkeratosis and papillomatosis are minimal-to-absent. H&E, ×400.

A 62-year-old Indian man with a strong family history of metabolic syndrome (MeTS) presented with asymptomatic, granular-surfaced, dark brown pigmentation on the cheeks (Figure 1, A and B). Nuchal/axillary acanthosis nigricans (AN) were conspicuously absent. Dermoscopy showed exaggerated light brown pseudoreticular network and scattered brown globules and structureless areas. At higher magnification (×150), oval dark brown annular “ring”-like structures centered around hair follicles were observed (Figure 1C). The dermoscopic image was conspicuously devoid of sulci/cristae (which are considered typical of AN). Examination and biochemical evaluation revealed blood pressure = 160/102 mm Hg, waist circumference = 92 cm, fasting blood glucose = 126 mg/dL, and fasting serum triglycerides = 438 mg/dL, confirming central obesity with MeTS. Histopathology revealed hypermelanized epidermis and dermis, and basal melanocytosis (Figure 1D). Clinicodermoscopicopathological correlation diagnosis was maturational hyperpigmentation (MH) with MeTS.

## Teaching Point

Although facial AN and MH may actually represent evolutionary standpoints on the morphological spectrum of cutaneous markers of MeTS (CMM) [1], labeling them as synonymous [2] without dermoscopy/supportive evidence seems inappropriate at present. Maturational hyperpigmentation is a newly described facial melanosis. It is a CMM akin to facial AN with which it does share some morphological features as well; however, specific features such as relatively softer surface with conspicuous but finer granularity and indistinct margins on gross morphology, and distinctive dermoscopy and histopathology [1], warrant identification of MH as a separate nosological entity. Thus, every physician’s awareness of this relatively less evident and lesser-known asymptomatic entity is paramount to facilitate prompt workup for MeTS.
